# Immunochemotherapy with Amphotericin B and HisAK70 Vaccine for Cutaneous Leishmaniosis

**DOI:** 10.3390/ijms27146181

**Published:** 2026-07-10

**Authors:** Socorro Espuelas, Carmen Palomino-Cano, Carlos Torrado-Salmerón, Helga K. Ruiz, Paloma M. de la Torre-Iglesias, Santiago Torrado-Santiago, Juan J. Torrado, José María Alunda, Christophe Dardonville, Sergio Alberto Sánchez Guirales, Dolores R. Serrano, Javier Carrión

**Affiliations:** 1 Department of Pharmaceutical Sciences, School of Pharmacy and Nutrition, University of Navarra, 31008 Pamplona, Spain; sespuelas@unav.es (S.E.); cpalominoca@unav.es (C.P.-C.); 2 Department of Pharmaceutics and Food Technology, Faculty of Pharmacy, Complutense University of Madrid, 28040 Madrid, Spain; ctorrado@ucm.es (C.T.-S.); helgakar@ucm.es (H.K.R.); pmtorre@ucm.es (P.M.d.l.T.-I.); torrado2@ucm.es (S.T.-S.); torrado1@farm.ucm.es (J.J.T.); sergsa16@ucm.es (S.A.S.G.); dr.serrano@farm.ucm.es (D.R.S.); 3 Instituto Universitario de Farmacia Industrial, Complutense University of Madrid, Plaza Ramón y Cajal s/n, 28040 Madrid, Spain; jmalunda@ucm.es; 4 Group ICPVet, Department of Animal Health, Faculty of Veterinary Medicine, Complutense University of Madrid, 28040 Madrid, Spain; 5 Institute of Medicinal Chemistry (IQM-CSIC), 28006 Madrid, Spain; dardonville@iqm.csic.es; 6 Research Institute Hospital 12 de Octubre, 28041 Madrid, Spain

**Keywords:** cutaneous leishmaniosis, immunochemotherapy, amphotericin B, therapeutic vaccine, HisAK70, drug repurposing, paromomycin, topical therapy, Th1 immune response

## Abstract

Cutaneous leishmaniosis (CL) remains a major neglected tropical disease, with current therapies constrained by toxicity, high cost, and variable efficacy. Here, we evaluated an immunochemotherapy strategy combining topical amphotericin B (AmB) with the therapeutic DNA vaccine HisAK70 in a murine model of *Leishmania major* infection. BALB/c mice were subcutaneously infected and treated with topical AmB cream alone, AmB plus HisAK70, or paromomycin (PM) as a reference therapy. Therapeutic efficacy was assessed through lesion progression, parasite burden in draining lymph nodes and spleen, and immunological markers associated with parasite control. Both PM and the combined AmB + HisAK70 treatment significantly reduced lesion progression and markedly decreased parasite burden compared with infected controls, demonstrating effective control of local infection and systemic dissemination. Importantly, the combination therapy enhanced the efficacy of AmB alone, supporting the beneficial contribution of vaccine-driven immune modulation to therapeutic outcome. Therapeutic efficacy was associated with reduced arginase activity in infected tissues and an increased IFN-γ/IL-4 ratio, indicative of a protective Th1-oriented immune response. Together, these findings highlight immunochemotherapy as a promising strategy for CL treatment, integrating localized topical drug delivery with targeted immune activation to improve therapeutic efficacy while potentially reducing systemic toxicity.

## 1. Introduction

The leishmanioses comprise a group of vector-borne diseases caused by protozoan parasites of the genus *Leishmania* and transmitted through the bite of infected phlebotomine sandflies. It is considered one of the most important neglected tropical diseases, affecting millions of people worldwide and placing more than one billion individuals at risk in endemic regions. The disease manifests in several clinical forms, including cutaneous, mucocutaneous and visceral leishmaniosis, each characterized by different clinical outcomes and epidemiological patterns. Among them, CL represents the most common presentation, characterized by localized ulcerative skin lesions [1].

CL is highly prevalent in tropical and subtropical regions and disproportionately affects rural and socioeconomically vulnerable populations, where environmental conditions favor sandfly transmission and access to healthcare remains limited. Recent global population displacement driven by armed conflicts, climate change, and socioeconomic instability has increased migration flows, contributing to the geographic redistribution of infectious diseases and the growing emergence of imported CL cases in Europe [2]. Although rarely fatal, CL imposes a considerable burden due to chronic lesions that often heal, leaving permanent scars. When located on visible areas such as the face, these scars can lead to significant consequences, impairing quality of life and social integration. This impact is particularly pronounced among women and girls, for whom disfiguring scars may reduce social acceptance, employment opportunities, and marriage prospects, often resulting in reduced self-esteem, social exclusion, and long-term psychological distress [3].

The management of CL remains challenging due to the limited availability of safe and effective treatments. Current therapeutic options include toxic and painful drugs such as pentavalent antimonials and AmB deoxycholate, less toxic but costly alternatives like liposomal AmB, and oral agents such as miltefosine, which remain associated with toxicity and limited accessibility in many endemic regions. In addition, local treatments such as cryotherapy or thermotherapy can be effective but also present important limitations [4]. Overall, existing therapies are frequently associated with toxicity, high cost, prolonged treatment regimens, and variable efficacy depending on parasite species and lesion characteristics, while many systemic drugs require hospital-based administration and careful monitoring of adverse effects, further restricting their accessibility in endemic areas [5]. These limitations have driven increasing interest in topical therapies as safer and more accessible alternatives for localized CL, with the potential to reduce systemic toxicity, improve treatment adherence, and lower healthcare costs [4,6]. They are currently recommended for uncomplicated CL, including patients with a limited number of small lesions, absence of lymphatic dissemination, and lesions located outside cosmetically or functionally sensitive areas. In more complex cases, topical treatments may also be used as adjuncts to systemic therapy to enhance local parasite clearance. However, despite these advantages, currently available local therapies remain suboptimal. Intralesional antimonials achieve cure rates of approximately 60–90% but require repeated painful injections and trained personnel, limiting their feasibility in endemic settings [7]. Topical paromomycin (PM), often combined with methylbenzethonium chloride and occasionally gentamicin, shows variable cure rates ranging from 50% to 80%, reflecting heterogeneity across parasite species and clinical contexts [8].

AmB has been extensively investigated as a topical treatment due to its potent antileishmanial activity [9]. However, its physicochemical limitations, including poor solubility and limited skin permeability, restrict effective dermal delivery despite the development of multiple formulations such as lipid-based systems, emulsions, hydrogels, and nanoparticle carriers. Clinical studies evaluating topical AmB formulations, including lipid-based preparations [6] and more recently cream formulations such as Anfoleish [10], have shown moderate and variable efficacy, highlighting the limited translation of potent in vitro activity into consistent clinical outcomes.

These challenges underscore the need for improved therapeutic strategies in CL. Combination approaches integrating antiparasitic drugs with host-directed therapies [11] are conceptually attractive, as parasite clearance ultimately depends on effective cellular immunity. A Th1-biased response, characterized by IFN-γ-mediated macrophage activation, is essential for intracellular parasite killing, suggesting that immunomodulation may enhance treatment efficacy while potentially reducing drug dosage and limiting the emergence of resistances [12].

In experimental studies using a hamster model of CL, the topical AmB cream formulation demonstrated partial reductions in both parasite burden and lesion progression [13]. The multi-antigenic DNA vaccine HisAK70 encodes several conserved *Leishmania* antigens, including nucleosomal histones, A2, KMP11, and HSP70, which are associated with parasite survival and virulence. This vaccine has been shown to induce Th1-type immune responses and confer cross-protection against different *Leishmania* species in experimental models [14].

In this context, the present study evaluated three therapeutic approaches in an experimental murine model of cutaneous leishmaniosis: (1) topical paromomycin (PM) as a reference treatment, (2) a topical amphotericin B (AmB) formulation solubilized with cyclodextrins and incorporated into a cream-based system, and (3) a combined immunochemotherapy strategy consisting of the same topical AmB formulation together with the therapeutic DNA vaccine HisAK70. Therapeutic efficacy was evaluated by measuring lesion progression, parasite burden in draining lymph nodes and spleen, and immunological markers associated with parasite control, including arginase activity and cytokine profiling.

## 2. Results

### 2.1. Therapeutic Efficacy Assessed by Lesion Progression

**Figure 1:**
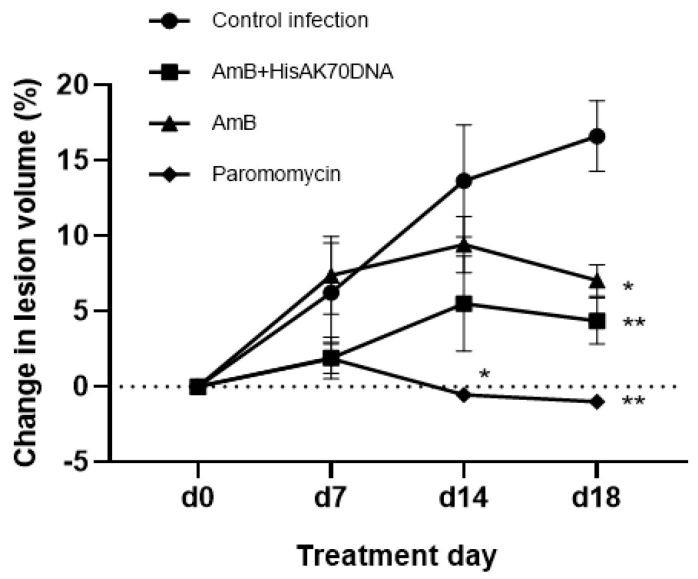
**Therapeutic efficacy evaluated by lesion progression in *L. major*-infected BALB/c mice.** Lesion development at the infection site located at the base of the tail was monitored during treatment in mice infected with *L. major*. Animals were treated with AmB cream, AmB cream combined with HisAK70 DNA (AmB + HisAK70 DNA), or paromomycin (PM), and compared with an infected untreated control group. Lesion size was measured using a digital caliper and expressed as change in lesion volume (%), calculated relative to baseline values at treatment initiation (day 27 post-infection pi). Negative values indicate lesion reduction relative to baseline, consistent with lesion healing. Data are presented as mean ± SEM (n = 6 mice per group). Statistical significance was determined using two-way ANOVA followed by Dunnett’s multiple comparisons test comparing each treatment group with the infected control at each time point. * *p* < 0.05; ** *p* < 0.01.

The therapeutic efficacy of the different treatments was evaluated by monitoring lesion progression in *Leishmania* major-infected BALB/c mice. Cutaneous lesions developed at the site of infection located at the base of the tail (distal dorsal region of the mice) and increased progressively in the untreated infected control group throughout the observation period (Figure 1).

At day 7 after treatment initiation (d7), no significant differences in lesion size were observed between treated groups and the infected control (Dunnett’s multiple comparisons test, *p* > 0.05), indicating that therapeutic effects were not yet detectable at this early stage. By day 14 (d14), mice treated with PM showed a significant reduction in lesion progression compared with the infected control group (mean difference = 14.19; 95% CI: 1.92–26.46; *p* = 0.0291). In contrast, the AmB group and the AmB + HisAK70 DNA combination group did not yet exhibit statistically significant differences relative to the control (*p* > 0.05). Additional multiple-comparison analyses performed at the end of treatment (day 18 post-treatment) revealed that PM-treated mice showed significantly lower lesion progression compared with the AmB-treated group, whereas no significant differences were observed between PM and the AmB + HisAK70 combination. These findings suggest that the combined therapy achieved a therapeutic effect comparable to the reference treatment during the late phase of lesion resolution.

**Figure 2:**
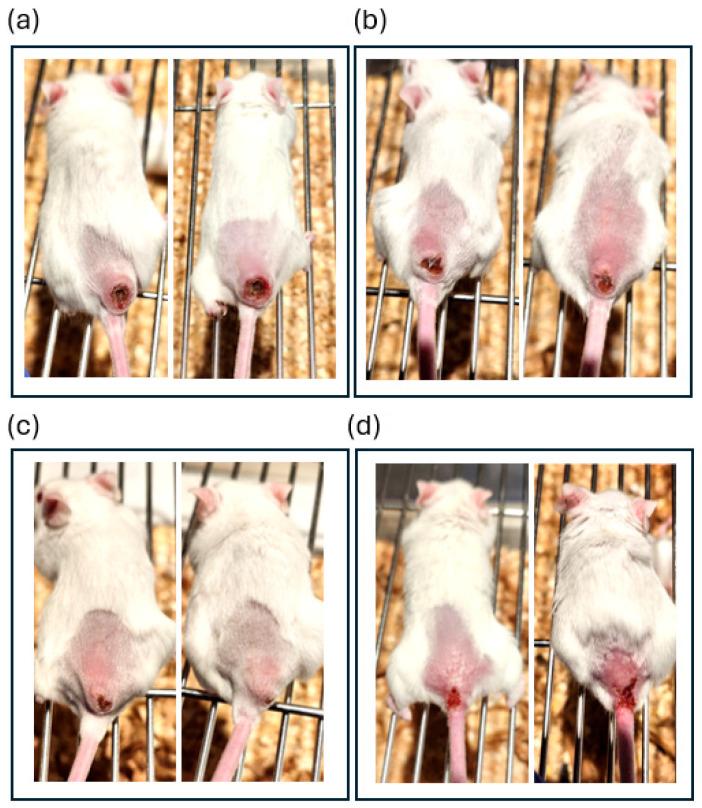
**Representative images of cutaneous lesions in *L. major*-infected BALB/c mice at day 18 of treatment.** Mice were assigned to the following experimental groups: (**a**) infected untreated control, (**b**) AmB cream, (**c**) AmB cream+ HisAK70 DNA, and (**d**) PM. Images show the lesion area at the base of the tail. Two representative animals per group are presented. The combination therapy exhibited a marked reduction in lesion size compared with AmB alone, supporting an enhanced therapeutic effect associated with the addition of the immunotherapeutic component. PM-treated mice showed pronounced lesion resolution accompanied by improved apparent tissue regeneration. Both treatments were clearly superior to the untreated control, consistent with quantitative lesion measurements. Images were acquired under identical conditions.

Representative images of lesion appearance at day 18 post-treatment further supported the quantitative findings (Figure 2).

Untreated infected mice exhibited large ulcerative lesions with marked visible tissue alteration at the infection site. In contrast, mice treated with AmB showed partial improvement, although lesions remained clearly detectable. More pronounced macroscopic improvement was observed in mice receiving the AmB + HisAK70 DNA combination, which displayed reduced lesion size and ulcerative appearance compared with untreated controls. PM-treated mice exhibited the most advanced lesion resolution, with markedly reduced visible lesion area and improved tissue appearance. These observations were consistent with the quantitative analysis of lesion progression, which revealed significant reductions in lesion size in the AmB-treated group (mean difference = 9.58; 95% CI: 1.91–17.24; * *p* < 0.05 (*p* = 0.0187)), the AmB + HisAK70 DNA combination group (mean difference = 12.26; 95% CI: 4.14–20.38; ** *p* < 0.01 (*p* = 0.0064)), and the PM-treated group (mean difference = 17.62; 95% CI: 9.88–25.36; ** *p* < 0.01 (*p* = 0.0016)) compared with infected untreated mice. Overall, therapeutic effects became more evident during the later stages of treatment, with the combination therapy achieving a therapeutic response comparable to the reference treatment.

### 2.2. Effects of Combined Therapy on Parasite Burden in Lymphoid Organs

**Figure 3:**
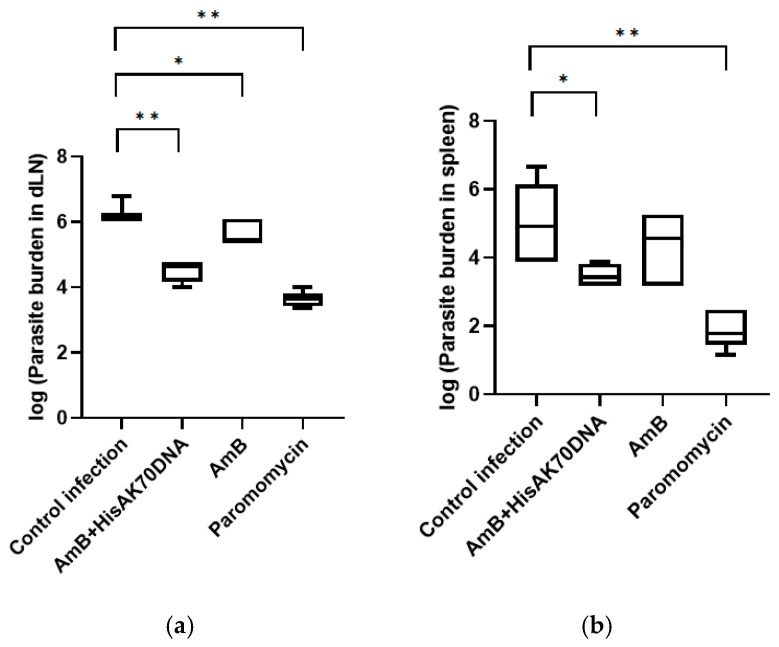
**Parasite burden in lymphoid organs of *L. major*-infected BALB/c mice.** (**a**) Parasite burden in draining inguinal lymph nodes (dLN); (**b**) Parasite burden in spleen tissue. Parasite load was determined at the experimental endpoint by limiting dilution assay and expressed as log parasite burden. Mice were treated with AmB cream, AmB cream combined with HisAK70 DNA (AmB + HisAK70 DNA), or PM and compared with an infected untreated control group. Data are presented as box plots showing median and interquartile range (n = 6 mice per group). Statistical significance was determined using one-way ANOVA followed by Dunnett’s multiple comparisons test versus the infected untreated control group and Tukey’s multiple comparisons test for pairwise comparisons between treatment groups. * *p* < 0.05; ** *p* < 0.01.

Parasite burden was determined at the experimental endpoint in both draining inguinal lymph nodes (dLN) and spleen using a limiting dilution assay (Figure 3).

In the draining lymph nodes (Figure 3a), untreated infected mice exhibited the highest parasite loads. All treatments significantly reduced parasite burden compared with the infected control group (* *p* < 0.05 or ** *p* < 0.01). AmB alone induced a moderate reduction in parasite burden, whereas the AmB + HisAK70 DNA combination produced a markedly greater decrease in parasite load (** *p* < 0.01), demonstrating the beneficial contribution of the immunotherapeutic component. Additional multiple-comparison analyses between treatment groups revealed significantly lower parasite levels in the combination group compared with AmB alone (** *p* < 0.01). PM treatment achieved the strongest reduction in local parasite burden (** *p* < 0.01); however, no significant differences were observed between PM and the combination therapy, indicating comparable efficacy in controlling parasite persistence within draining lymphoid tissue. Systemic parasite dissemination was evaluated by quantifying parasite burden in the spleen (Figure 3b). The AmB + HisAK70 DNA combination significantly reduced splenic parasite burden compared with the infected control group (* *p* < 0.05), whereas treatment with AmB alone did not achieve a statistically significant reduction. PM treatment resulted in the most pronounced decrease in splenic parasite load (** *p* < 0.01). Nevertheless, no significant differences were detected between PM and the combination therapy, further supporting the capacity of the combined treatment to effectively control systemic parasite dissemination.

When parasite burden was expressed as a percentage reduction relative to the infected control group, the AmB + HisAK70 DNA combination achieved a 98.1% reduction in the draining lymph nodes and a 99.6% reduction in the spleen. AmB alone produced a moderate reduction in parasite burden, particularly in lymph nodes (72%), while showing a more limited overall effect. As expected, PM treatment produced the highest reductions in parasite burden, reaching 99.8% in draining lymph nodes and complete parasite clearance in the spleen (100%).

### 2.3. Modulation of Arginase Activity in Lesion Tissue

**Figure 4:**
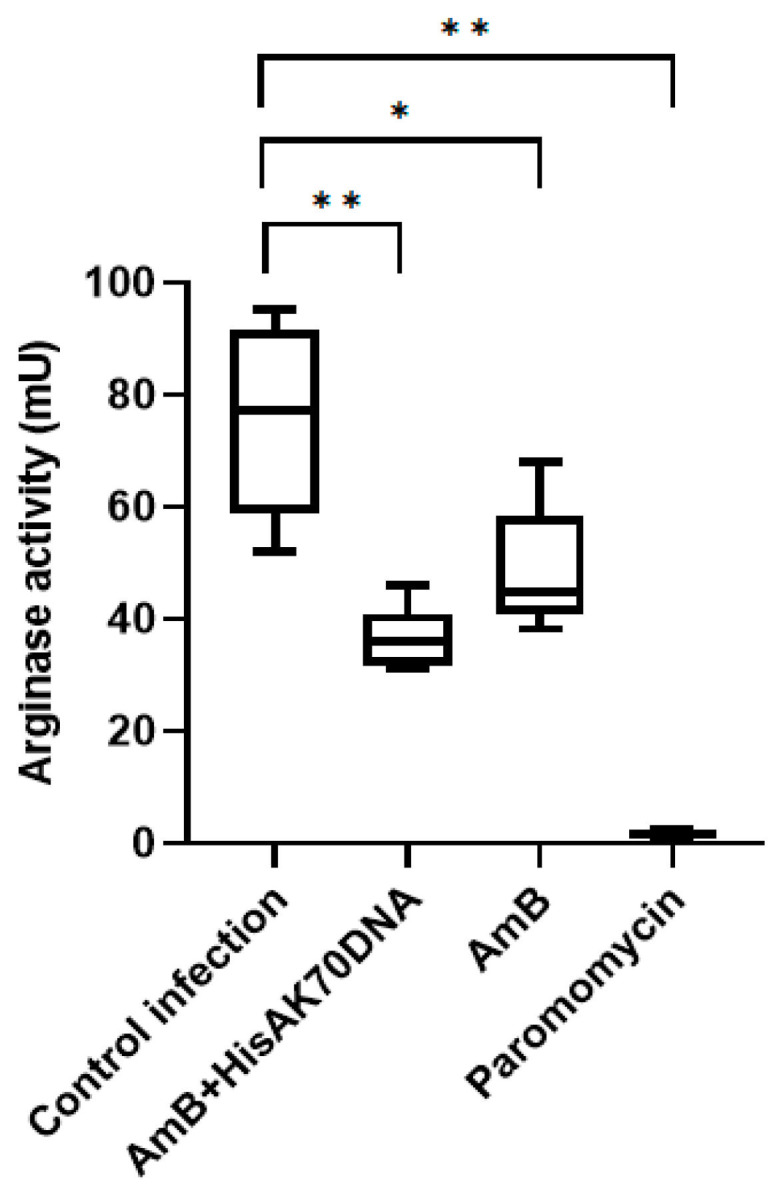
**Arginase activity in lesion tissue of *L. major*-infected BALB/c mice.** Arginase activity was measured in lesion biopsies collected at the experimental endpoint and expressed as milliunits (mU) of enzymatic activity. Mice were treated with AmB cream, AmB cream combined with HisAK70 DNA (AmB + HisAK70 DNA), or PM and compared with an infected untreated control group. Data are presented as box plots showing median and interquartile range. Statistical significance was determined using one-way ANOVA followed by Dunnett’s multiple comparisons test versus the infected untreated control group and Tukey’s multiple comparisons test for pairwise comparisons between treatment groups. * *p* < 0.05; ** *p* < 0.01.

Arginase activity was evaluated in lesion tissue at the experimental endpoint as an indicator of macrophage activation status (Figure 4).

Untreated infected mice exhibited the highest arginase activity levels, consistent with the establishment of a permissive inflammatory microenvironment during Leishmania major infection. All treatment groups showed reduced arginase activity compared with infected controls, although the magnitude of reduction varied among therapies. The AmB + HisAK70 DNA combination significantly decreased arginase activity relative to the untreated infected group (* *p* < 0.05), while AmB alone induced a more moderate reduction. PM-treated mice displayed the lowest arginase activity values (** *p* < 0.01), indicating a stronger modulation of the lesion-associated inflammatory response. Additional multiple-comparison analyses between treatment groups did not reveal significant differences between the AmB + HisAK70 combination and AmB alone. These findings suggest that the reduction in arginase activity may be primarily associated with decreased parasite burden and improvement of the inflammatory microenvironment at the lesion site rather than with a specific additive effect of the immunotherapeutic component on arginase regulation.

### 2.4. Cytokine Balance in Draining Lymph Nodes

**Figure 5:**
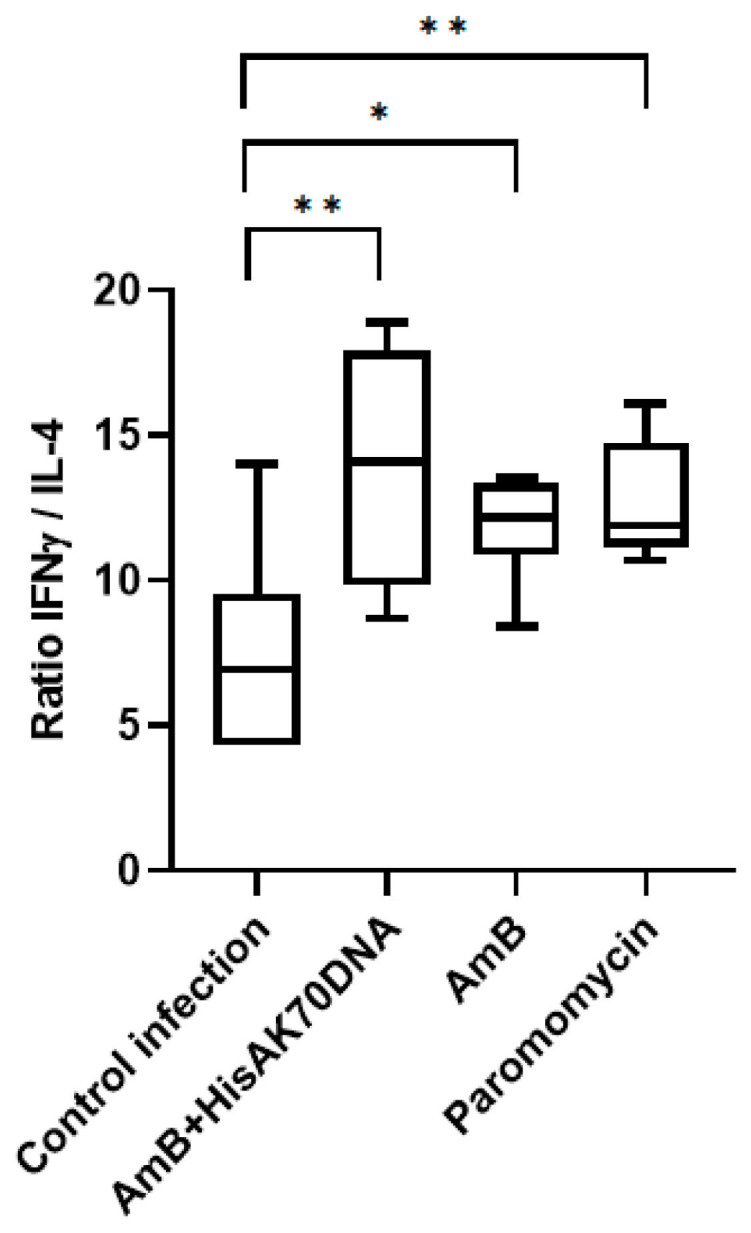
**IFN-γ/IL-4 ratio in draining lymph node cultures following SLA stimulation**. Draining lymph node cells from *L. major*-infected BALB/c mice were co-cultured with dendritic cells pulsed with soluble *Leishmania* antigen (SLA). The ratio of IFN-γ to IL-4 production was calculated from cytokine levels measured in culture supernatants. Mice were treated with AmB cream, AmB cream combined with HisAK70 DNA (AmB + HisAK70 DNA), or PM and compared with an infected untreated control group. Data are presented as box plots showing median and interquartile range. Statistical significance was determined using one-way ANOVA followed by Dunnett’s multiple comparisons test versus the infected control group. * *p* < 0.05, ** *p* < 0.01.

To further evaluate the immunological effects associated with treatment, cytokine production was analyzed in draining lymph node cell cultures stimulated with soluble *Leishmania* antigen (SLA). The IFN-γ/IL-4 ratio was subsequently calculated as an indicator of Th1/Th2 immune balance (Figure 5).

Untreated infected mice exhibited relatively low IFN-γ/IL-4 ratios, consistent with the susceptibility profile typically associated with progressive L. major infection in BALB/c mice. In contrast, mice treated with the AmB + HisAK70 DNA combination showed a significant increase in the IFN-γ/IL-4 ratio compared with infected controls (* *p* < 0.05), indicative of a shift toward a protective Th1-oriented immune response. AmB alone and PM treatment also displayed increased IFN-γ/IL-4 ratios relative to untreated controls, although no statistically significant differences were observed among treatment groups. These findings suggest that the combined therapy promotes a more favorable immunological environment associated with parasite control, supporting the contribution of the HisAK70 immunotherapeutic component to the induction of Th1-biased antileishmanial responses.

## 3. Discussion

The present study evaluated an immunochemotherapeutic strategy combining topical AmB with the HisAK70 DNA vaccine and compared its efficacy with AmB monotherapy and topical PM, one of the most effective topical treatments currently available for CL. PM produced the greatest reduction in lesion progression, whereas the combination therapy improved therapeutic outcomes relative to AmB alone and achieved parasite burdens and immune responses in the draining lymph node comparable to those observed with PM (Figure 1, Figure 3 and Figure 5). In contrast, AmB monotherapy showed limited efficacy and did not significantly reduce parasite burden.

Lesion progression reflects processes occurring locally within infected tissue. In experimental models of CL, lesion size is associated with parasite burden but also integrates local inflammatory responses and tissue remodeling [15,16]. In this study, PM induced a faster and more pronounced reduction in lesion size than combination therapy, suggesting more effective local parasite control and/or a greater impact on inflammation-driven tissue damage. The superior efficacy of PM compared with AmB can be explained by their physicochemical and pharmacological properties. As a hydrophilic aminoglycoside, PM is expected to be distributed efficiently within inflamed cutaneous tissue, facilitating early exposure of infected macrophages and accumulation at effective concentrations [17]. In contrast, AmB, although intrinsically more potent in vitro, is highly lipophilic, extensively protein-bound, and predominantly membrane-associated, with activity largely restricted to the unbound fraction. These characteristics probably reduce its bioavailability after topical administration and delay therapeutic activity [18]. In addition, anti-inflammatory effects have been reported for PM, which may further contribute to improved lesion resolution [19].

Arginase activity at the site of infection followed a pattern consistent with lesion outcomes (Figure 4). As a functional biomarker frequently associated with alternatively activated macrophages and parasite persistence, reduced arginase activity is generally indicative of a tissue environment less permissive to parasite survival. Because arginase activity was measured only at the experimental endpoint, the observed differences most likely reflect treatment outcome rather than a primary determinant of efficacy [20,21,22,23]. Nevertheless, because arginase activity may derive from multiple cellular sources within the lesion microenvironment, these results should not be interpreted as direct evidence of macrophage polarization. However, total arginase activity remains a widely recognized functional biomarker associated with susceptibility to *Leishmania* infection, parasite persistence, and treatment response in experimental models of CL.

In contrast, parasite burden and immune responses measured in the draining lymph node indicated that therapeutic vaccination enhanced parasite control relative to AmB alone. One limitation of the present study is that a HisAK70-alone treatment group was not included. Therefore, although the combined treatment clearly improved therapeutic outcomes relative to AmB monotherapy, the experimental design does not allow a formal distinction between additive and synergistic interactions between the two therapeutic components. Future studies incorporating vaccine-only treatment groups will be required to further characterize the nature of this interaction. However, this improvement was not accompanied by greater lesion resolution, suggesting that local drug activity remains a major determinant of clinical outcome. This apparent dissociation suggests that immune responses generated in the draining lymph node may not fully translate into effective parasite control at the site of infection, potentially due to constraints imposed by the local tissue microenvironment on effector function, similar to what has been described in tumor microenvironments [24]. The limited correlation between immune responses in the draining lymph node and treatment efficacy may also reflect the restricted interpretative value of the IFN-γ/IL-4 ratio. Although these cytokines represent key markers of Th1/Th2 polarization in experimental L. major infection, this simplified metric does not fully capture the complexity of the immune response in CL. In particular, regulatory mediators such as IL-10 may limit macrophage activation despite the presence of IFN-γ, thereby reducing effective parasite killing [25,26,27]. Future studies evaluating a broader cytokine panel will provide a more comprehensive characterization of the immune mechanisms associated with therapeutic efficacy. Moreover, additional cytokines involved in macrophage activation and Th1 polarization, including IL-12 and TNF-α, were not evaluated in the present study and could provide a more comprehensive characterization of the mechanisms underlying vaccine-induced immune modulation. Future studies aimed at optimizing this immunochemotherapeutic strategy should therefore incorporate broader cytokine profiling and more detailed immunological analyses.

The therapeutic schedule was selected on the basis of previous studies performed by our group with the HisAK70 DNA vaccine against different *Leishmania* species, which indicated that vaccine-induced immunomodulatory responses become established approximately 10–15 days after subcutaneous administration. Therefore, the first vaccine dose was administered shortly before initiation of topical AmB treatment with the aim of promoting a more favorable immune environment during the therapeutic phase, potentially facilitating the subsequent antiparasitic activity of the drug. A second vaccine dose was administered 10 days later to reinforce and sustain this response throughout treatment. Although this rationale was based on previous experience with the vaccine, immune kinetics were not specifically evaluated in the present study. Future studies should characterize cytokine and cellular responses throughout treatment to optimize immunochemotherapeutic schedules. The HisAK70 vaccine was administered subcutaneously near the draining lymph nodes because its therapeutic objective is not to exert direct antileishmanial activity, but rather to induce a parasite-specific immune response capable of enhancing host-mediated parasite control. In contrast to amphotericin B, which acts directly on parasites at the lesion site, HisAK70 was intended to stimulate adaptive immune mechanisms through antigen presentation and T-cell activation within the regional lymphoid compartment, a key site for the initiation of protective immunity against *Leishmania* infection. Thus, the selected administration route was intended to complement the direct leishmanicidal activity of topical AmB with systemic immunomodulation. Nevertheless, alternative routes such as perilesional or intralesional administration may further enhance local immune activation and deserve evaluation in future studies.

Parenteral AmB in combination with vaccines has previously been evaluated in murine models of visceral leishmaniosis [28,29,30]. This is the first report combining topical AmB administration with a DNA vaccine in a murine model of CL. Although substantial differences (parenteral and visceral), co-administration strategies generally enhance antileishmanial efficacy through complementary mechanisms: the drug reduces parasite burden, while the vaccine elicits a Th1/M1 immune response capable of promoting parasite killing [31]. In agreement with these findings, clinical studies in humans have also reported beneficial effects in CL following the combination of the adjuvanted polyprotein vaccine LEISH-F1 + MPL-SE with meglumine antimoniate, further supporting the translational potential of immunochemotherapy approaches for leishmaniosis treatment [32]. Because animals were euthanized at day 45 pi according to predefined welfare criteria, the present study does not allow evaluation of long-term parasite clearance, relapse rates, or the durability of treatment-induced protection. Future studies should therefore incorporate extended follow-up periods and assessment of vaccine-induced immunological memory.

Taken together, these findings suggest that therapeutic outcome is strongly influenced by effective parasite reduction at the site of infection and largely driven by the direct antiparasitic activity of the drug. Although the combination therapy improved outcomes relative to AmB alone, it did not outperform PM, which consistently achieved the strongest control of lesion progression in this model. Nevertheless, the combination strategy introduces a complementary mechanism based on immune-mediated parasite control, as reflected by enhanced immune activation in the draining lymph node. While local drug activity appears to be a major contributor to lesion resolution, immune modulation may be particularly advantageous under conditions associated with suboptimal treatment outcomes, including incomplete response, relapse, or impaired host immunity. In this context, the use of AmB is especially attractive because its mechanism of action, based on ergosterol binding and membrane destabilization, has been associated with a relatively low frequency of resistance development in *Leishmania* parasites compared with other antileishmanial drugs [33]. By contrast, resistance to paromomycin has been more readily described and appears to involve multifactorial adaptive mechanisms affecting several parasite pathways [34]. Since paromomycin interferes with ribosomal function and protein synthesis, alterations in translational machinery and parasite metabolism may facilitate adaptation under sustained drug pressure [33,34]. These observations suggest that, despite their high efficacy, therapeutic strategies based on paromomycin combinations may carry a greater risk of resistance selection than AmB-based approaches. Therefore, combining topical AmB with immunotherapeutic strategies such as HisAK70 may represent a promising and durable therapeutic alternative for CL. Although PM achieved the strongest effect on lesion resolution in the present study, AmB was selected as the immunotherapeutic partner because of its distinct mechanism of action, favorable resistance profile, and the availability of a previously optimized topical formulation. The superior efficacy of PM observed in this model highlights its value as a reference topical treatment for CL. Nevertheless, the improved outcomes obtained with the AmB + HisAK70 combination support the concept that immunomodulatory strategies can enhance the efficacy of conventional antileishmanial drugs. Future studies should evaluate whether similar or even greater benefits may be achieved by combining HisAK70 with PM or other topical antileishmanial agents.

## 4. Materials and Methods

### 4.1. Experimental Design

**Figure 6:**
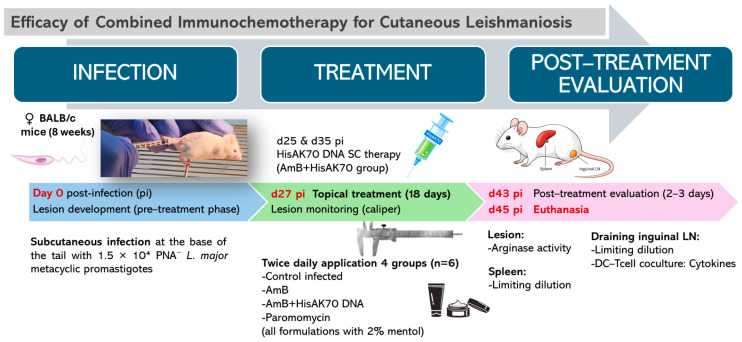
**Experimental design of the murine model of cutaneous leishmaniosis and treatment protocol.** BALB/c mice were subcutaneously infected at the base of the tail with 1.5 × 10^4^ PNA^−^ *L. major* metacyclic promastigotes. Lesion development was monitored during the pre-treatment phase. Mice received topical treatment twice daily for 18 days with AmB cream, AmB cream combined with HisAK70 DNA immunotherapy, or PM, while infected untreated mice served as controls. Post-treatment evaluation included lesion monitoring, parasite burden determination in lesion tissue, draining inguinal lymph nodes and spleen, arginase activity analysis, and cytokine profiling.

The experimental workflow, including infection, treatment administration, and post-treatment analyses, is summarized in Figure 6.

### 4.2. Animals

Female BALB/c mice (8 weeks old) were purchased from Charles River Laboratories and maintained under specific pathogen-free conditions with ad libitum access to food and water. Animals were housed under controlled environmental conditions (22 ± 2 °C, 12 h light/dark cycle).

The experimental design, including the sample size (n = 6 animals per group), was established based on previous experience with this murine model of cutaneous leishmaniasis and in accordance with the principle of reduction within the 3Rs framework for animal experimentation. All procedures were conducted in accordance with institutional and national guidelines for animal welfare and were approved by the Ethics Committee of the Complutense University of Madrid (PROEX 048.2/24).

Mice were randomly allocated to treatment groups. Investigators were not formally blinded during lesion measurements or parasite burden determination; however, all outcome variables were assessed using predefined objective criteria. Animals were monitored regularly throughout the study for lesion progression, ulceration, body condition, body weight, and signs of discomfort or distress. Humane endpoints included excessive lesion progression, severe ulceration, marked weight loss, or any clinical condition compromising animal welfare. The experimental endpoint was established at day 45 pi, as lesions in untreated control animals were expected to progress toward ulceration beyond this stage. No animals reached the predefined humane endpoints before the planned termination of the experiment.

### 4.3. Parasites and Infection

*L. major* (clone V1: MHOM/IL/80/Friedlin) promastigotes were cultured at 26 °C in Schneider’s insect medium supplemented with 10% heat-inactivated fetal bovine serum (FBS) and an antibiotic mixture under standard conditions.

Parasites were maintained until the stationary phase, and metacyclic promastigotes were enriched by negative selection using peanut agglutinin (PNA^−^).

Female BALB/c mice were subcutaneously infected at the base of the tail with 1.5 × 10^4^ PNA^−^ metacyclic promastigotes suspended in 50 µL of sterile phosphate-buffered saline (PBS). The day of parasite inoculation was designated as day 0 pi.

Lesion development was monitored weekly using a digital caliper by measuring lesion length, width, and induration to estimate lesion volume. Monitoring began on day 27 pi, when visible cutaneous lesions were present in all infected animals and therapeutic intervention was initiated.

### 4.4. Treatment Regimen

At day 27 pi, when cutaneous lesions were clearly established in all animals, mice were randomly assigned (n = 6 per group) to the following experimental groups: (i) infected untreated control, (ii) AmB cream, (iii) AmB cream combined with HisAK70 DNA (AmB + HisAK70 DNA), and (iv) PM, used as a reference antileishmanial treatment.

Topical formulations were applied twice daily for 18 consecutive days (from day 27 to day 43 pi). A total of 50 mg of formulation was applied per lesion at each administration. All topical formulations were prepared using a vehicle containing 2% menthol to minimize removal by grooming behavior and to enhance transdermal drug permeation. The AmB formulation consisted of a 0.05% AmB–γ-cyclodextrin inclusion complex incorporated into a Lanette N cream base, with purified water as vehicle. The PM formulation consisted of an oil-in-water emulsion with 15% drug (Cetyl alcohol 1.5%, Stearic acid 2.5%, menthol 2%, Petrolatum 5%, liquid paraffin 10%, Glyceryl monostearate 11% and water 70%). In the combination therapy group, HisAK70 DNA (100 µg per dose) was administered subcutaneously on days 25 and 35 pi as part of the immunotherapeutic protocol. The dose was distributed bilaterally in the lateral flanks near the inguinal draining lymph nodes. The experimental endpoint was established at day 45 pi, two days after completion of treatment.

### 4.5. Determination of Arginase Activity, Parasite Burden, and Cytokine Production

At day 45 pi, blood samples were collected for serum isolation and mice were subsequently euthanized. An incisional skin biopsy from the lesion site (approximately 3–4 mm × 2–3 mm × 1–2 mm) was obtained for determination of arginase activity. Draining inguinal lymph nodes (dLN) and spleens were aseptically collected for parasite burden determination and cytokine analysis.

Arginase activity in lesion tissue was determined as an indicator of macrophage activation status. Skin biopsies (~50 mg) were collected from the infection site at the experimental endpoint, homogenized, and arginase activity was quantified by measuring urea production from L-arginine using a colorimetric microassay. Enzymatic activity was measured according to the micromethod described by Corraliza et al. and later adapted for *Leishmania* infection models [22,35]. Urea production was measured spectrophotometrically after incubation of tissue lysates with L-arginine substrate, and arginase activity was expressed as milliunits (mU) of enzymatic activity.

Parasite burdens in draining inguinal lymph nodes and spleens were determined by limiting dilution culture as previously described [36]. Briefly, organs were harvested aseptically and homogenized in 1 mL of Schneider’s insect medium (Sigma-Aldrich, St. Louis, MO, USA) supplemented with 10% heat-inactivated fetal calf serum (FCS), penicillin, and streptomycin. Homogenates were subjected to four-fold serial dilutions in 96-well culture plates containing the same medium and incubated at 26 °C. For each animal, organ homogenates were analyzed in quadruplicate using independent limiting dilution culture series, and parasite burden was calculated from the mean value obtained.

After 7 days of incubation, wells were examined under an inverted phase-contrast microscope to detect viable *L. major* promastigotes. Parasite burden was determined as the highest dilution showing parasite growth and expressed as log parasite load per organ.

To evaluate antigen-specific cytokine responses, draining inguinal lymph node cells were isolated at the experimental endpoint and processed into single-cell suspensions under sterile conditions. One week prior to sacrifice, bone marrow-derived dendritic cells (DCs) from naïve mice were generated and pulsed with soluble *Leishmania* antigen (SLA; 50 µg/mL) as described elsewhere [37]. On the day of sacrifice, lymph node cells were washed and resuspended at a final concentration of 3 × 10^6^ cells/mL in complete Dulbecco’s modified Eagle’s medium (DMEM) supplemented with 10% heat-inactivated fetal calf serum (FCS), 2 mM L-glutamine, 100 U/mL penicillin, and 100 µg/mL streptomycin. Cells were plated at 1 mL per well in 24-well plates and co-cultured with SLA-pulsed DCs at a T cell:DC ratio of 5:1 at 37 °C in a humidified atmosphere containing 5% CO_2_. After 72 h of incubation, culture supernatants were collected and cytokine levels were quantified by enzyme-linked immunosorbent assay (ELISA) according to the manufacturers’ instructions. IFN-γ and IL-4 were measured, and the IFN-γ/IL-4 ratio was calculated to assess the balance between Th1 and Th2 immune responses. Cytokine concentrations were determined in duplicate for each individual animal sample, and mean values were used for subsequent analyses.

### 4.6. Statistical Analysis

Lesion progression was expressed as the percentage change in lesion volume relative to baseline values at the start of treatment (day 27 pi) and calculated according to the following formula, where *Vₜ* represents the lesion volume at a given time point and *V_baseline_* corresponds to the lesion volume at treatment initiation:

$$\Delta Volume\left(\%\right)=\frac{V_{t}-V_{baseline}}{V_{baseline}}\times 100$$

Negative values indicate a reduction in lesion size relative to baseline, consistent with apparent lesion healing.

Data are presented as mean ± SEM unless otherwise indicated. Statistical analyses were performed using GraphPad Prism software version 8.0.2 (GraphPad Software, San Diego, CA, USA). Differences in lesion progression between groups over time were evaluated using two-way analysis of variance (ANOVA) followed by Dunnett’s multiple comparisons test, comparing each treatment group with the infected untreated control at each time point. For parasite burden, arginase activity, and cytokine ratio analyses, differences between groups were assessed using one-way ANOVA followed by Dunnett’s multiple comparisons test versus the infected untreated control group and Tukey’s multiple comparisons test for pairwise comparisons between treatment groups, as indicated in the corresponding figure legends. A *p*-value < 0.05 was considered statistically significant, with significance levels indicated as follows: * *p* < 0.05; ** *p* < 0.01.

## 5. Conclusions

The immunochemotherapeutic strategy combining topical amphotericin B (AmB) with the HisAK70 DNA vaccine demonstrated greater therapeutic efficacy than AmB alone for the control of experimental *L. major* infection. The combined therapy significantly improved lesion control, reduced parasite burden, and promoted a protective Th1-oriented immune response, supporting the potential of immunochemotherapy as an effective strategy for CL. Importantly, the use of AmB is particularly attractive due to its mechanism of action based on ergosterol binding and membrane destabilization, which has been associated with a relatively low propensity for resistance development compared with other antileishmanial drugs, including paromomycin. These findings further highlight the potential of drug repurposing and innovative combination therapies for CL. Nevertheless, several important challenges remain. As this study was performed in a murine model, further validation in advanced experimental and clinically relevant systems is required to confirm both efficacy and safety, particularly regarding drug permeation, tissue regeneration, and long-term therapeutic benefit. In addition, optimization of dosing regimens and formulation design may further enhance therapeutic outcomes. Importantly, because infection control and tissue repair are tightly interconnected, a deeper understanding of the interactions between immune cells and *L. major* parasites will be essential for the successful clinical translation of combined therapeutic strategies. This is especially relevant for CL, a disease that disproportionately affects vulnerable, stigmatized, and resource-limited populations where safe, effective, and accessible treatments remain urgently needed. Taken together, our findings position immunochemotherapy as a promising and translatable therapeutic paradigm for CL, integrating topical drug delivery with targeted immune modulation to achieve more effective, safer, and potentially more accessible treatment strategies.

## Data Availability

The original contributions presented in this study are included in the article. Further inquiries can be directed to the corresponding author.

## Abbreviations

The following abbreviations are used in this manuscript:

CL	Cutaneous leishmaniosis
AmB	Amphotericin B
PM	Paromomycin
dLN	Draining lymph node
SLA	Soluble Leishmania antigen
IFN-γ	Interferon gamma
IL-4	Interleukin 4
Th1	T helper 1
Th2	T helper 2
SEM	Standard error of the mean
PNA	Peanut agglutinin
